# Chinese herbal foot baths as a new strategy for diabetic foot with Wagner grade of 0 or 1: a meta-analysis and data mining

**DOI:** 10.3389/fphar.2025.1594386

**Published:** 2025-07-10

**Authors:** Ruifang Lin, Yunfeng Yu, Yuman Yin, Xiu Liu, Yongjun Wu, Qin Xiang, Yaoyue Luo, Rong Yu

**Affiliations:** ^1^ School of Traditional Chinese Medicine, Hunan University of Chinese Medicine, Changsha, Hunan, China; ^2^ School of Pharmacy, Hunan University of Chinese Medicine, Changsha, Hunan, China; ^3^ School of Nursing, Hunan University of Chinese Medicine, Changsha, Hunan, China

**Keywords:** Chinese herbal, foot-bath, diabetic foot, Wagner grade of 0 or 1, meta-analysis, data mining

## Abstract

**Objective:**

The benefit of Chinese herbal foot-baths in treating diabetic foot remains unclear. This study aims to assess the efficacy of Chinese herbal foot-baths for diabetic foot with Wagner grade of 0 or 1 and identify key candidate herbs.

**Methods:**

A comprehensive search of eight databases was conducted for studies published up to 24 May 2025. Relevant data on study characteristics, outcomes, and risk of bias were extracted. The meta-analysis and trial sequential analysis (TSA) were performed using RevMan 5.3 and TSA 0.9.5.10 beta, respectively. The risk ratio (RR) and mean difference (MD) were respectively used as effect sizes for dichotomous and continuous outcomes. Publication bias was assessed with funnel plots and Egger’s tests.

**Results:**

13 studies involving 921 participants were included in this review. The meta-analysis showed that compared to warm water foot-bath, Chinese herbal foot-baths significantly improved the clinical effective rate (RR 1.42, 95%CI 1.31–1.53, *p* < 0.00001), ankle-brachial index (MD 0.19, 95%CI 0.11–0.26, *p* < 0.00001), common peroneal nerve motor nerve conduction velocity (MD 4.09, 95%CI 2.41–5.77, *p* < 0.00001), common peroneal nerve sensory nerve conduction velocity (MD 3.83, 95%CI 2.48–5.17, *p* < 0.00001). The glycosylated hemoglobin A1c (MD -0.15, 95%CI -0.30–0.01, *p* = 0.04), and fasting blood glucose levels (MD -0.28, 95%CI -0.54–0.02, *p* = 0.04) were significantly reduced. However, no significant differences were observed in 2-h postprandial blood glucose, total cholesterol, triglycerides, and adverse events (*p* > 0.05). Additionally, except for the clinical effective rate, there are no potential publication biases in other results. Furthermore, the data mining identified the key candidate herbs used in the foot bath as [*Cinnamomum cassia* Presl], [*Conioselinum anthriscoides* ‘Chuanxiong'], [*Paeonia lactiflora* Pall.], [*Angelica sinensis* (Oliv.) Diels] [*Prunus persica* (L.), Batsch], [*Carthamus tinctorius* L.], and [*Asarum heterotropoides* F. Schmidt].

**Conclusion:**

Chinese herbal foot-baths can improve clinical symptoms as well as vascular and nerve functions in diabetic foot patients with Wagner grades 0 or 1, without increasing the incidence of adverse events. The seven herbs identified through data mining offer a reference for formulating Chinese herbal foot-baths. However, these clinical findings and the pharmacological effects of the herbal combinations require further validation.

**Systematic Review Registration:**

www.crd.york.ac.uk/PROSPERO/view/CRD42024615181, CRD42024615181

## 1 Introduction

Diabetes and its complications are the main reasons threatening human health (Li Y. et al., 2023; Zhao et al., 2023; An and Ren, 2024; Liu et al., 2024; Zuo et al., 2024). Diabetic foot is a severe lower limb infection caused by diabetic peripheral nerve and vascular disease (Lin et al., 2021). Epidemiological studies indicate that diabetic patients have a lifetime risk of 25% for developing diabetic foot, which is increasing annually (Jodheea-Jutton et al., 2022). Diabetic foot is characterized by symptoms such as ulcers, osteomyelitis, osteoarticular destruction, and gangrene, which are one of the main causes affecting the patients’ daily life (Lin et al., 2021). At the same time, it is also the most important cause of hospitalization and lower limb amputation in diabetic patients (Volmer-Thole and Lobmann, 2016; Brocco et al., 2018). It is reported that about 67% of amputations are related to diabetes every year in the United States, and this value is as high as 90% in the United Kingdom (Ahmad et al., 2016). Early identification of foot lesions and timely treatment of foot ulcers are key to the management of diabetic foot, which often requires the participation of multiple disciplines (Xia et al., 2019). Although treatments such as surgical debridement, oxygen therapy, negative pressure wound therapy, biological agents, and growth factors have slowed the progression of diabetic foot, their exorbitant costs have brought a huge economic burden to individuals, families, and society (Xia et al., 2019; Akkus and Sert, 2022). The National Health Service in the United Kingdom reportedly spends approximately £1 billion on diabetic foot ulcers, with the majority of costs related to hospital admissions (Ahmad et al., 2016). Therefore, it is of great significance to explore a safe, effective, and affordable auxiliary treatment for diabetic foot.

**FIGURE 1:**
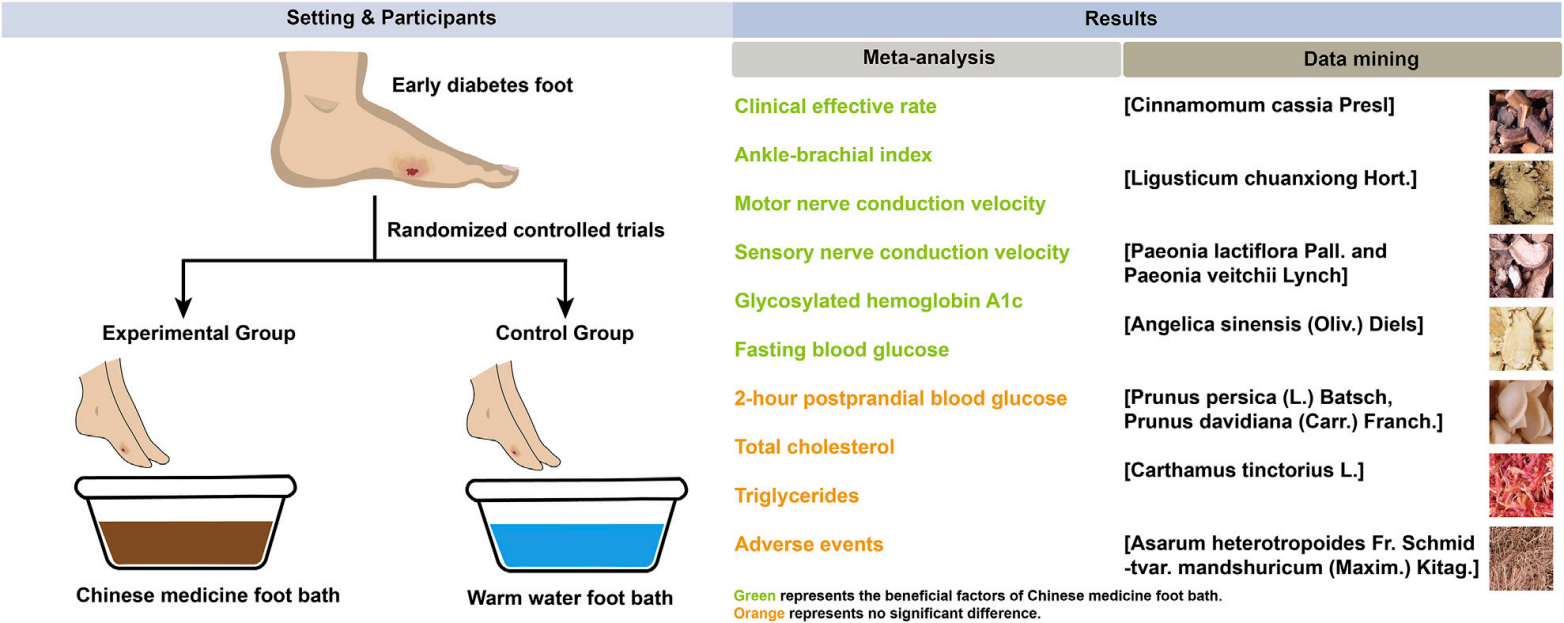
Graphical abstract.

With the advancement of research, the efficacy of Chinese herbal medicine in treating diabetes (Xu et al., 2025) and its complications (Zhang et al., 2024a), including diabetic cardiomyopathy (Li et al., 2022; 2024), diabetic nephropathy (Zhou et al., 2023; Zhang et al., 2024b; Zhao et al., 2024; Chung et al., 2023), diabetic peripheral neuropathy (Jo et al., 2023), and diabetic erectile dysfunction (Wang et al., 2024a), has been extensively verified. The Chinese herbal foot bath is a unique therapeutic method that uses decoction of Chinese herbal medicine to fumigate and wash the feet. It allows the active ingredients of Chinese herbal medicine to penetrate into the skin through warm water fumigation and washing, thereby exerting the effect of on various ailments (Du, 2023). The Chinese herbal foot bath has the characteristics of easy operation, safety, and reliability (Lv and Luo, 2023), and is widely used in China for the treatment of colds, fevers, or peripheral neuropathy (Lin et al., 2020; Gu and Wang, 2022). Recent clinical trials have demonstrated its potential in alleviating early symptoms associated with diabetic foot and promoting blood circulation in the foot (Zhang and Sun, 2023). However, there is no meta-analysis and data mining of Chinese herbal foot baths for early diabetic foot, and their specific effects and prescription of Chinese herbal medicine remain unclear. In this study, we used meta-analysis to evaluate the benefits and risks of Chinese herbal foot baths compared with warm water foot baths in the treatment of diabetic foot with Wagner grade of 0 or 1, and used data mining to explore the prescription of Chinese herbal medicine for foot baths. The design and findings of this study are shown in Figure 1.

## 2 Methods

This study strictly followed the Preferred Reporting Items for Systematic reviews and Meta-Analyses (PRISMA) (Page et al., 2021) and was registered in PROSPERO (CRD42024615181). We conducted the first search on 30 November 2024 and updated the search on 24 May 2025.

### 2.1 Literature search

The literature was searched using a combination of subject terms and extra terms. The search formula was: ((Foot Bath OR Fumigation OR Fumigations OR Washing OR Steaming OR Lavipeditum) AND (Diabetic Foot OR Diabetic Feet OR Diabetes Foot OR Diabetes Feet)). Relevant studies were retrieved from four English-language databases (Embase, PubMed, Cochrane Library, and Web of Science) and four Chinese-language databases (China National Knowledge Infrastructure [CNKI], China Science and Technology Journal Database [VIP], Wanfang Data, and Sinomed). Additionally, gray literature was explored by searching the World Health Organization International Clinical Trials Registry Platform (WHO-ICTRP) and ClinicalTrials.gov. References from relevant reviews were also reviewed to ensure comprehensive coverage. The search was current up to 24 May 2025, with no language restrictions. The detailed search strategies for each database used in the initial search are provided in Supplementary Table S1.

### 2.2 Inclusion and exclusion criteria

Inclusion criteria: (i) Participants were required to meet the diagnostic standards for T2DM as specified by the WHO in 1999, as well as the diagnostic criteria for diabetic foot established by any national or international authoritative organization such as the Chinese Guidelines for the Diagnosis and Treatment of Diabetic Foot (The Diabetic Foot Branch of the China Healthcare International Exchange Promotion Association, 2017) and Traditional Chinese Medicine Standards for Diabetic Foot Diagnosis and Treatment (Fan et al., 2011). Additionally, participants needed to be classified as Wagner grade 0 or 1. (ii) The control group received a warm water foot bath, while the experimental group received Chinese herbal foot baths. (iii) The primary efficacy outcomes included the clinical effective rate, common peroneal nerve sensory nerve conduction velocity (SNCV), common peroneal nerve motor nerve conduction velocity (MNCV), and ankle-brachial index (ABI). The clinical effective rate was assessed using a symptom scale based on the *Guidelines for Clinical Research of New Traditional Chinese Medicine* (Zheng, 2002). A reduction of 30% or more in the symptom scale score was defined as effective. The clinical effective rate refers to the proportion of participants rated as effective relative to the total number of participants, used to evaluate the improvement of diabetic foot-related symptoms (Zheng, 2002). The ABI was used to assess blood circulation in the lower limbs, with a decrease indicating insufficient blood flow and an increased risk of foot ulcers (Brito-Zurita et al., 2013). Common peroneal nerve SNCV and MNCV were measured to evaluate the sensory and motor functions of the peroneal nerve, with decreases indicating the extent of nerve damage (Lawrence and Locke, 1961). Secondary efficacy outcomes include blood glucose levels (hemoglobin A1c [HbA1c], fasting blood glucose [FBG], and 2-h postprandial blood glucose [PBG]) and lipid levels (total cholesterol [TC] and triglycerides [TG]). HbA1c provides an average of blood glucose levels over the past 2–3 months, serving as a marker of long-term blood glucose control, while FBG and PBG reflect immediate control of fasting and postprandial blood glucose levels, respectively. TC and TG are indicators of lipid metabolism, which are particularly important in managing diabetic complications. The safety outcome was defined as the adverse events. (iv) The study design was a randomized controlled trial (RCT).

Exclusion criteria: (i) Duplicate publication of data. (ii) Incomplete data. (iii) Data not available.

### 2.3 Literature screening

The literature screening process began with the organization of all retrieved articles using Endnote X9. A systematic approach was employed to select studies based on predefined inclusion and exclusion criteria. These criteria were established to ensure that only relevant studies addressing the research question were included. Each article was carefully reviewed for eligibility, focusing on aspects such as study design, population characteristics, and intervention details. The screening process was conducted in multiple stages, including title and abstract screening followed by full-text review.

### 2.4 Data collection

Once the relevant studies were identified, essential data were compiled into a comprehensive table using Excel 2010. This table included critical information such as the primary author’s name, publication year, sample sizes, male-to-female ratio, average age of participants, course of disease, Wagner grade, frequency of foot baths, duration of treatment, and various clinical parameters. The clinical parameters collected encompassed the clinical effective rate, ABI, MNCV, SNCV, HbA1c, FBG, 2h-PBG,TC, TG, and adverse events. This structured data collection process ensured that all relevant information was systematically organized for further analysis.

### 2.5 Risk assessment of bias

Risk of Bias 2 (RoB 2) tool was used to assess the risk of bias in each included study (Sterne et al., 2019), which evaluates five specific domains: the randomization process, deviations from intended interventions, missing outcome data, outcome measurement, and selection of reported results. The risk of bias for each domain was classified as low, high, or of some concern according to the Cochrane Handbook for Systematic Reviews of Interventions (Sterne et al., 2019). This thorough risk assessment aimed to provide a clear understanding of the quality and reliability of the included studies. The above assessment was conducted independently by two reviewers, and any disagreement was resolved through discussion and adjudication by a third reviewer.

### 2.6 Statistical analysis

The meta-analysis was conducted using RevMan 5.3 software. Effect measures for continuous variables were represented as the mean difference (MD), while the risk ratio (RR) and the Peto-odds ratio (PetoOR) were utilized for dichotomous variables. Heterogeneity among studies was assessed using the I^2^ statistic, with I^2^ values interpreted as follows: I^2^ < 50% indicated low to moderate heterogeneity, while I^2^ ≥ 50% suggested high heterogeneity. Given the inherent variability in interventions, particularly with individualized treatments such as polyherbal prescriptions, random-effect models were applied for all analyses to account for this variability.

To explore potential sources of heterogeneity, meta-regression analyses were conducted when the number of included studies was greater than 10 to ensure sufficient statistical power and reliability. These analyses aimed to assess the impact of covariates such as baseline characteristics, treatment duration, or intervention specifics on outcomes with significant heterogeneity. If the number of studies was insufficient (n ≤ 10), meta-regression was not performed, and heterogeneity was instead explored via sensitivity analyses. Sensitivity analyses were performed to evaluate the robustness of the meta-analysis results and to explore potential sources of heterogeneity. This involved a leave-one-out approach, where each individual study was sequentially excluded from the pooled analysis. The impact of each exclusion was assessed by noting changes in the overall effect size, confidence interval (CI), and heterogeneity metrics. A significant reduction in heterogeneity after removing a specific study suggested that this study might be a primary contributor to heterogeneity. Additionally, if the exclusion of any individual study did not materially alter the overall effect estimate, the results were considered robust and stable. This process helped confirm the reliability of the findings and contributed to understanding the consistency of the evidence. Subgroup analyses were performed based on clinical parameters such as Wagner grade, treatment frequency, treatment duration, and the composition and dosage of Chinese herbal medicine, with the aim of investigating the impact of clinical heterogeneity on the primary outcome of clinical effective rate.

The TSA was conducted to assess the robustness of cumulative evidence in the meta-analysis while controlling for type I and II errors from repeated significance testing. TSA was performed using version 0.9.5.10 beta, with a two-sided alpha of 5% and 80% power. The required information size was calculated based on the anticipated effect size, control event rate, and heterogeneity-adjusted diversity. A cumulative Z-curve was plotted against predefined monitoring boundaries to determine whether the evidence was sufficient to confirm or refute the intervention effect. If the Z-curve crossed the boundaries before reaching the required sample size, the evidence was considered conclusive; otherwise, further trials were likely needed.

Funnel plots were created to visually assess publication bias by examining the symmetry of effect sizes against their standard errors; any asymmetry may indicate potential bias. To enhance this visual assessment, Egger’s tests were performed to statistically quantify asymmetry by regressing the standard normal deviate (effect size divided by standard error) against precision (inverse of the standard error). A significant result (*p* < 0.05) suggests possible publication bias. By combining the visual evaluation from the funnel plot with the results from Egger’s test, we aimed to more accurately assess the likelihood of publication bias in the meta-analysis.

### 2.7 Certainty of evidence

The certainty of the evidence for outcomes was assessed using the Grading of Recommendations, Assessment, Development, and Evaluation (GRADE) approach, considering factors such as risk of bias, inconsistency, indirectness, imprecision, and publication bias. This process was performed independently by two reviewers, with disagreements resolved through discussion. We used the GRADEpro Guideline Development Tool (gradepro.org) to facilitate transparent and consistent ratings. Randomized trials started as high certainty, and adjustments were made based on the evaluation of each domain. The final ratings were categorized as high, moderate, low, or very low certainty, providing a clear measure of confidence in the evidence supporting our findings.

### 2.8 Data mining

Frequency analysis and association rule analysis were performed using SPSS Modeler 18.0 to identify key candidate herbs (Wang et al., 2024b; 2024c). In the frequency analysis, a threshold of ≥30% was established to pinpoint high frequency herbs utilized in foot-bath treatments. This threshold signifies that at least 30% of the analyzed cases included a specific herb, underscoring its prevalent use within the dataset (Yu et al., 2025). Subsequently, these high frequency herbs would be used for association rule analysis.

For the association rule analysis, the *Apriori* model was employed. The parameters were set with a support threshold of ≥30%, a confidence threshold of ≥80%, and a lift threshold of ≥1.0 to investigate key candidate herbs combinations. The support threshold represents the proportion of transactions in the dataset that include both the antecedent and consequent items, thereby indicating how frequently these items co-occur. The confidence threshold measures the likelihood that the consequent occurs given the presence of the antecedent, reflecting the strength of the association. Meanwhile, the lift threshold assesses the degree of independence between the antecedent and consequent, with lift >1.0 indicating a positive correlation. These thresholds were selected based on established practices in the literature (Yu et al., 2024b; 2024a), which indicate that a support level of 30% ensures that the identified associations are not merely coincidental, while a confidence level of 80% suggests a strong likelihood of the consequent occurring when the antecedent is present. The herbs identified within these combinations are defined as key candidate herbs.

## 3 Results

### 3.1 Literature screening

**FIGURE 2:**
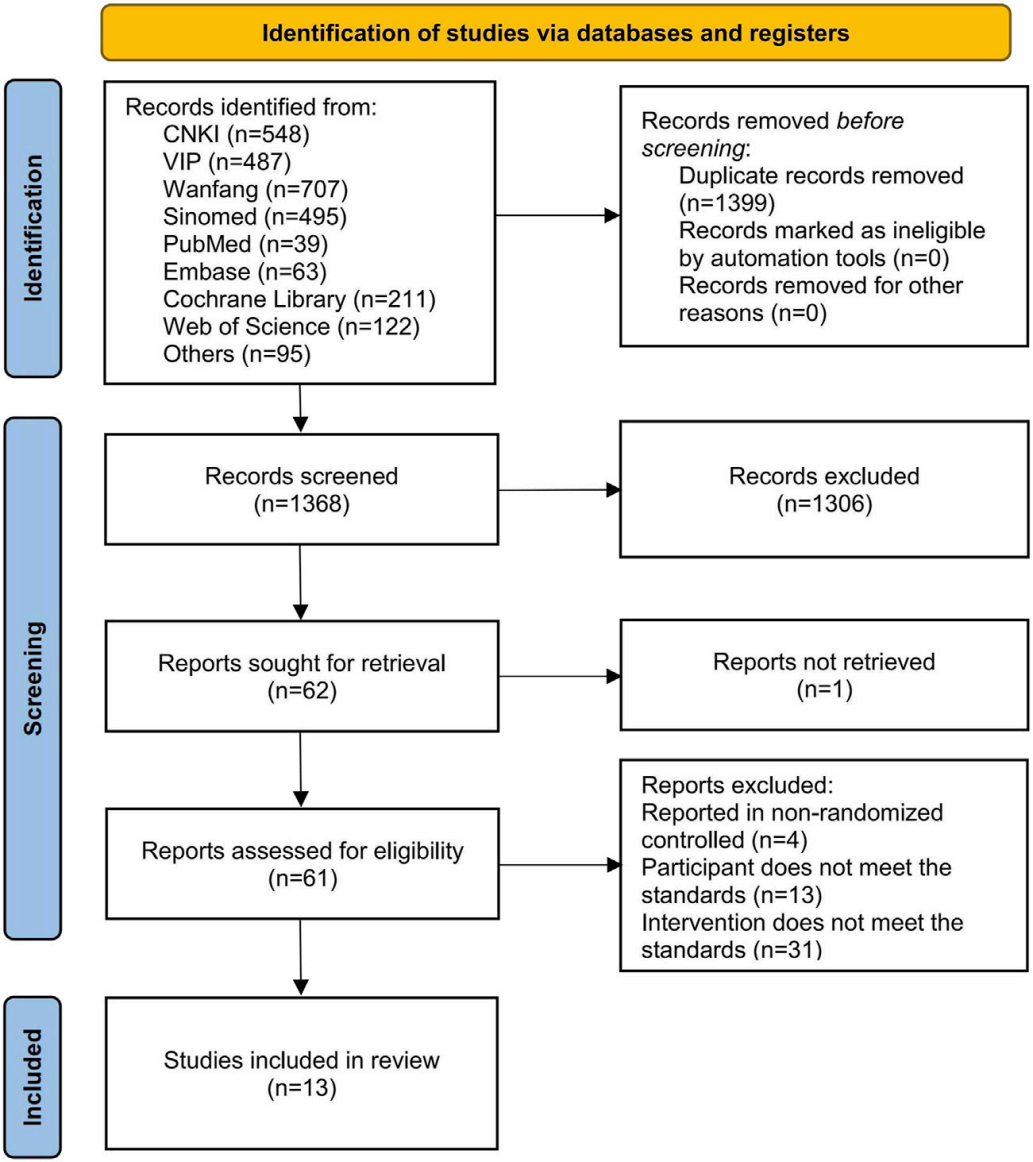
Flowchart of literature screening.

A total of 2,767 articles were identified from various databases, including 548 from CNKI, 487 from VIP, 707 from Wanfang, 495 from Sinomed, 39 from PubMed, 63 from Embase, 211 from the Cochrane Library, 122 from Web of Science, 42 from ClinicalTrials.gov, 46 from WHO ICTRP, and 7 from reference lists. During the screening process, 1,399 articles were eliminated due to duplication, and additional 1,355 articles were excluded for failing to meet the inclusion criteria. Ultimately, 13 articles (Gao, 2009; Zhang et al., 2011; Zhou et al., 2011; Su, 2012; Chen and Zhao, 2014; Huang, 2014; Huang and Wang, 2015; Zhou et al., 2015; Ding and Huang, 2016; Liu, 2016; Gao, 2018; Jiang et al., 2018; Zhang et al., 2018) were included in this study, as shown in Figure 2.

### 3.2 Basic characteristics of included studies

A total of 13 clinical studies (Gao, 2009; Zhang et al., 2011; Zhou et al., 2011; Su, 2012; Chen and Zhao, 2014; Huang, 2014; Huang and Wang, 2015; Zhou et al., 2015; Ding and Huang, 2016; Liu, 2016; Gao, 2018; Jiang et al., 2018; Zhang et al., 2018) were included in this review, all of which were conducted in China between 2009 and 2018, encompassing a total sample size of 921 participants. Among these, 451 participants underwent warm water foot baths, while 470 received Chinese herbal foot baths (Table 1). The composition and dosage of the Chinese herbs used in the included studies are detailed in Supplementary Table S2.

**TABLE 1:** Basic characteristics of the included studies.

Study	Sample (E/C)	Male/%	Age/years	Course of disease /years	Wagner	Foot-bath frequency	Course of treatment/days	Chinese herbals
Chen and Zhao (2014)	37/36	50.7	57.0	9.2	0	30 min bid	60	[*Arnebia euchroma* (Royle ex Benth.) I.M.Johnst.], [*Angelica sinensis* (Oliv.) Diels], [*Angelica dahurica* (Fisch. ex Hoffm.) Benth. & Hook. f. ex Franch. & Sav.], [*Glycyrrhiza uralensis* Fisch.]
Ding and Huang (2016)	45/45	57.8	60.5	7.6	0	30 min qd	14	[*Asarum heterotropoides* F.Schmidt], [*Conioselinum anthriscoides* ‘Chuanxiong’], [*Achyranthes bidentata *Blume], [*Angelica dahurica* (Fisch. ex Hoffm.) Benth. & Hook. f. ex Franch. & Sav.], [*Cinnamomum cassia* Presl], [*Aconitum kusnezoffii* Rchb.], [*Clematis chinensis* Osbeck], [*Saposhnikovia divaricata* (Turcz. ex Ledeb.) Schischk.], [*Heracleum hemsleyanum* Diels], [*Aucklandia lappa* Decne.]
Gao (2018)	47/47	52.1	56.0	3.3	0	30 min qd	28	[*Trachelospermum jasminoides* (Lindl.) Lem.], [*Spatholobus suberectus* Dunn], [*Piper kadsura* (Choisy) Ohwi], [*Uncaria rhynchophylla* (Miq.) Miq.], [*Clematis chinensis* Osbeck], [*Carthamus tinctorius* L.], [*Liquidambar formosana* Hance], [*Salvia miltiorrhiza* Bunge], [*Prunus persica* (L.) Batsch], [*Aconitum carmichaelii* Debeaux], [*Asarum heterotropoides* F.Schmidt], [*Cinnamomum cassia* Presl]
Gao (2009)	35/35	45.7	66.7	9.9	0	20 min qd	60	[*Angelica sinensis* (Oliv.) Diels], [*Conioselinum anthriscoides* 'Chuanxiong'], [*Paeonia lactiflora* Pall.], [*Rehmannia glutinosa* (Gaertn.) Libosch. ex DC.], [*Prunus persica* (L.) Batsch], [*Carthamus tinctorius* L.], [*Cinnamomum cassia* Presl], [*Pheretima aspergillum* (E. Perrier)], [*Salvia miltiorrhiza* Bunge], [*Corydalis yanhusuo* (Y.H.Chou & Chun C.Hsu) W.T.Wang ex Z.Y.Su & C.Y.Wu]
Huang (2014)	20/20	60.0	58.8	/	0-1	30 min qd	14	[*Speranskia tuberculata* (Bunge) Baill.], [*Conioselinum anthriscoides* ‘Chuanxiong’], [*Carthamus tinctorius* L.], [*Boswellia serrata* Roxb.], [*Commiphora myrrha* (T.Nees) Engl.], [*Salvia miltiorrhiza* Bunge]
Huang and Wand (2015)	38/36	62.2	63.5	9.1	0	20 min bid	28	[*Phellodendron chinense* C.K.Schneid.], [*Sanguisorba officinalis* L.], [*Portulaca oleracea* L.], [*Taraxacum mongolicum* Hand.-Mazz.], [*Viola philippica* Cav.], [*Houttuynia cordata* Thunb.], [*Lobelia chinensis* Lour.], [KAl(SO₄)₂·12H₂O and NH₄Al(SO₄)₂·12H₂O], [*Artemisia argyi* Levl. et Vant.], [*Zanthoxylum schinifolium* Sieb. et Zucc.]
Jiang et al. (2018)	18/20	44.7	64.7	8.3	0-1	30 min bid	60	[*Prunus persica* (L.) Batsch], [*Carthamus tinctorius* L.], [*Rehmannia glutinosa* (Gaertn.) Libosch. ex DC.], [*Paeonia lactiflora* Pall.], [*Paeonia lactiflora* Pall.], [*Angelica sinensis* (Oliv.) Diels], [*Conioselinum anthriscoides* 'Chuanxiong'], [*Cinnamomum cassia* Presl], [*Bupleurum chinense* DC.], [*Bupleurum scorzonerifolium* Willd.], [*Pseudocydonia sinensis* (Dum.Cours.) C.K.Schneid.], [*Boswellia serrata* Roxb.]
Liu (2016)	30/30	53.3	50.6	11.8	0	30 min qd	90	[*Whitm.ania Pigra* Whitmania], [*Paeonia lactiflora* Pall.], [*Angelica sinensis* (Oliv.) Diels], [*Prunus persica* (L.) Batsch], [*Rheum palmatum* L.], [*Rehmannia glutinosa* (Gaertn.) Libosch. ex DC.], [*Pueraria montana* var. lobata (Willd.) Maesen & S.M.Almeida ex Sanjappa & Predeep], [*Anemarrhena asphodeloides* Bunge]
Su (2012)	28/28	/	/	/	0-1	20 min bid	28	[*Angelica sinensis* (Oliv.) Diels], [*Rehmannia glutinosa* (Gaertn.) Libosch. ex DC.], [*Conioselinum anthriscoides* 'Chuanxiong'], [*Paeonia lactiflora* Pall.], [*Achyranthes bidentata *Blume], [*Speranskia tuberculata* (Bunge) Baill.], [*Cinnamomum cassia* Presl], [*Asarum heterotropoides* F.Schmidt], *Aconitum carmichaeli* Debeaux, [*Aconitum kusnezoffii* Rchb.], [*Boswellia serrata* Roxb.], [*Commiphora myrrha* (T.Nees) Engl.], [*Pinellia ternata* (Thunb.) Makino], [*Atractylodes lancea* (Thunb.) DC.], [*Ephedra sinica* Stapf], [*Sinapis alba* L.]
Zhang et al. (2011)	50/50	29.0	61.9	10.9	0-1	20 min q2d	84	[*Astragalus mongholicus Bunge*], [*Prunus persica* (L.) Batsch], [*Carthamus tinctorius* L.], [*Gastrodia elata* Blume], [*Artemisia argyi* Levl. et Vant.], [*Cinnamomum cassia* Presl], [*Anemarrhena asphodeloides* Bunge], [*Phellodendron chinense* C.K.Schneid.], [*Saposhnikovia divaricata* (Turcz. ex Ledeb.) Schischk. ], [*Cnidium monnieri* (L.) Cuss.]
Zhang et al. (2018)	30/30	48.3	65.6	5.8	0	30 min qd	28	[*Spatholobus suberectus* Dunn], [*Heracleum hemsleyanum* Diels], [*Taxillus sutchuenensis* (Lecomte) Danser], *Gentiana macrophylla* Pall.], [*Conioselinum anthriscoides* 'Chuanxiong'], [*Eucommia ulmoides* Oliv.], [*Achyranthes bidentata *Blume], [*Dipsacus asper* Wall. ex DC.], [*Saposhnikovia divaricata* (Turcz. ex Ledeb.) Schischk.], [*Cinnamomum cassia* Presl], [*Pheretima aspergillum* (E. Perrier)], [*Asarum heterotropoides* F.Schmidt]
Zhou et al. (2011)	62/44	54.7	58.0	17.8	1	20 min qd	84	[*Rheum palmatum* L.], [*Phellodendron chinense* C.K.Schneid.], [*Calvatia lilacina* (Mont. et Berk.) Lloyd.], [KAl(SO₄)₂·12H₂O and NH₄Al(SO₄)₂·12H₂O], [*Ilex pubescens* Hook. & Arn.], [*Cinnamomum cassia* Presl]
Zhou et al. (2015)	30/30	55.0	65.3	4.7	0	30 min qd	28	[*Piper kadsura* (Choisy) Ohwi], [*Trachelospermum jasminoides* (Lindl.) Lem.], [*Uncaria rhynchophylla* (Miq.) Miq.], [*Spatholobus suberectus* Dunn], [*Clematis chinensis* Osbeck], [*Liquidambar formosana* Hance], [*Prunus persica* (L.) Batsch], [*Carthamus tinctorius* L.], [*Salvia miltiorrhiza* Bunge], [*Cinnamomum cassia* Presl], [*Aconitum carmichaelii* Debeaux], [*Asarum heterotropoides* F.Schmidt]

### 3.3 Risk of bias

**FIGURE 3:**
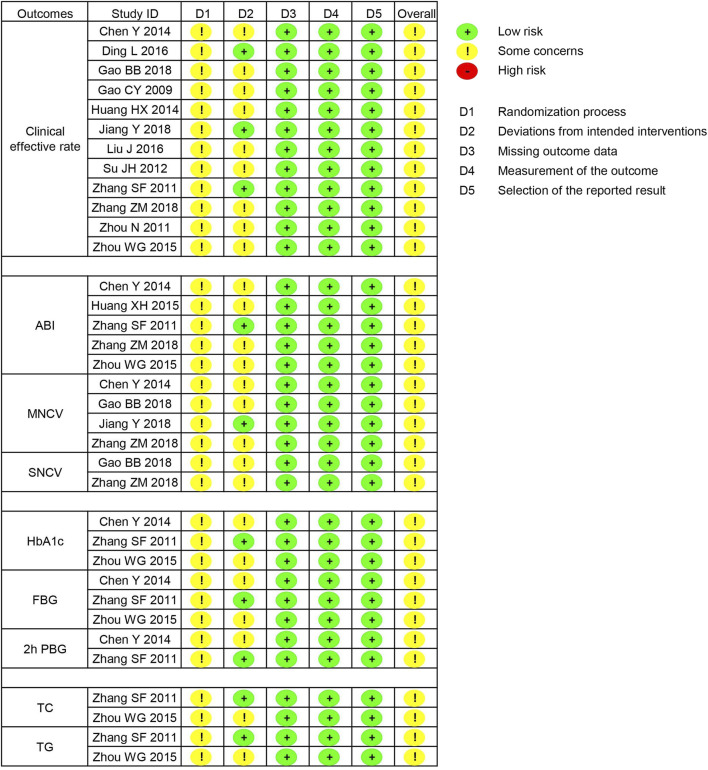
Risk of bias summary.

In the randomization process, seven studies utilized random number tables, while six studies did not report details regarding the random sequence generation; however, all studies did not provide information on allocation concealment. Therefore, 13 studies were all assessed as having some concerns regarding the randomization process. In the deviations from intended interventions, three studies employed a placebo design, while 10 studies did not describe the blinding details. Consequently, 10 studies were rated as having some concerns in this area. Additionally, all studies reported dropout rates of less than 20%, and the outcome measurements were not influenced by blinding, with all pre-specified outcomes reported. Therefore, the domains of missing outcome data, measurement of the outcome, and selection of the reported result for all studies were assessed as low risk. In summary, the overall risk of bias for the 13 studies was rated as some concerns, as shown in Figure 3.

### 3.4 Meta-analysis

#### 3.4.1 Clinical effective rate

**FIGURE 4:**
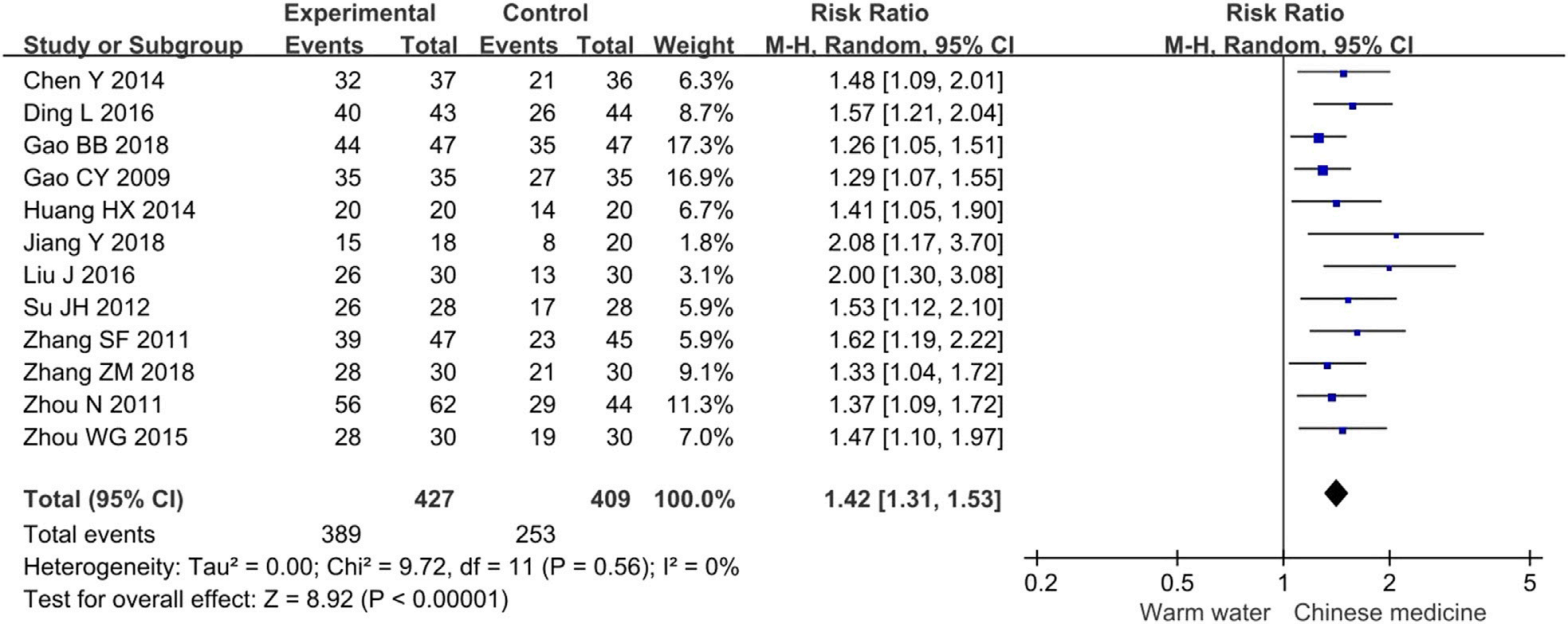
Forest plots of meta-analysis on the clinical effective rate.

The meta-analysis of the clinical effective rate included 12 studies involving 836 participants. The results indicated that, compared to warm water foot baths, Chinese herbal foot baths significantly increased the clinical effective rate by 42% for diabetic foot patients with Wagner grades of 0 or 1 (RR 1.42, 95% CI 1.31 to 1.53, *p* < 0.00001, I^2^ = 0%), as shown in Figure 4. This means that patients receiving Chinese herbal foot baths are 1.42 times more likely to experience relief from clinical symptoms compared to those receiving warm water foot baths. Furthermore, the sensitivity analysis confirmed the robustness of these findings regarding the clinical effective rate.

#### 3.4.2 Vascular and nerve function

##### 3.4.2.1 ABI

**FIGURE 5:**
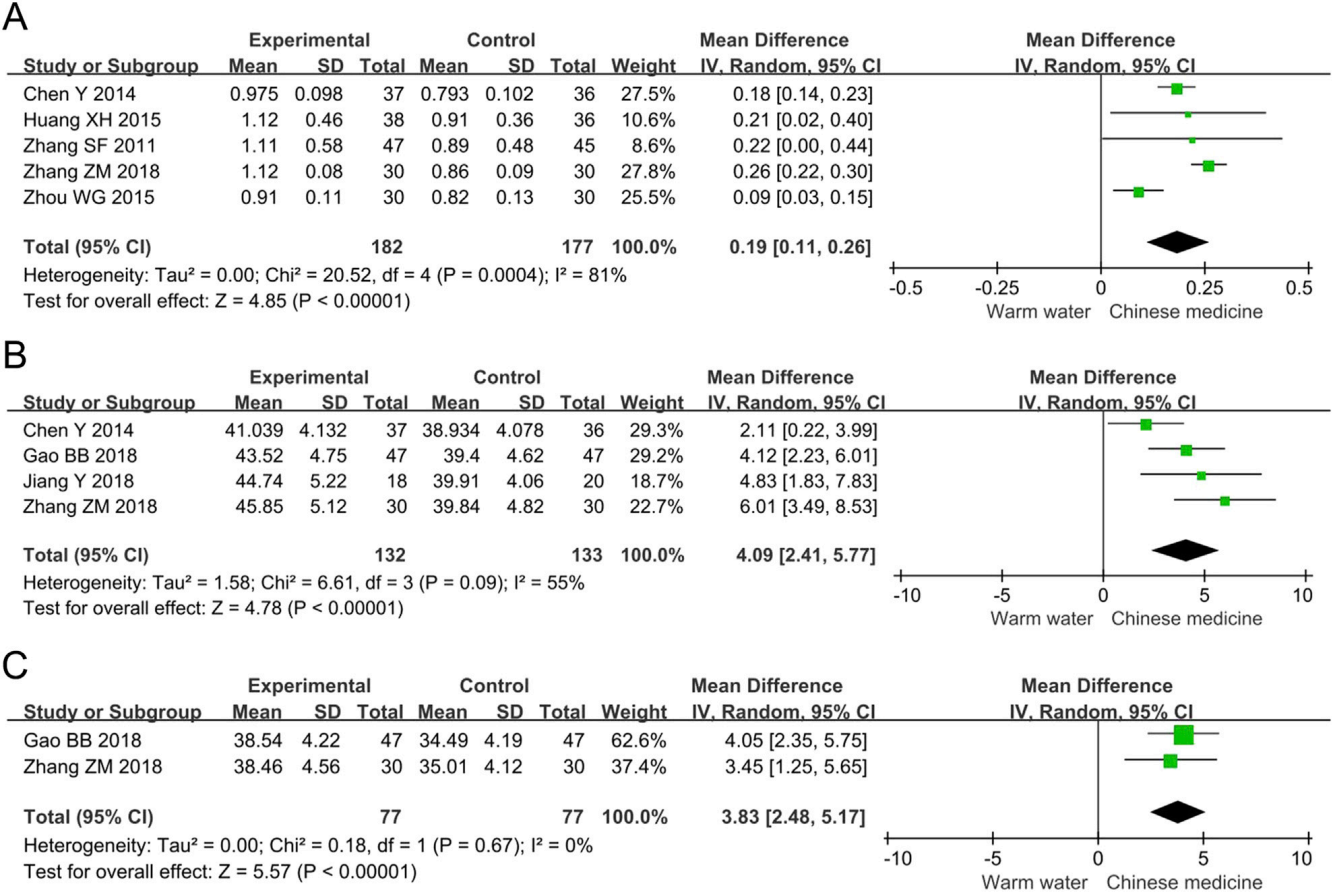
Forest plots of meta-analysis on the vascular and nerve function: **(A)** ABI; **(B)** Common peroneal nerve MNCV; **(C)** Common peroneal nerve SNCV. ABI, ankle-brachial index; MNCV, motor nerve conduction velocity; SNCV, sensory nerve conduction velocity.

The meta-analysis of ABI included six studies involving 399 participants. The results indicated that, compared to warm water foot baths, Chinese herbal foot baths significantly increased ABI by 0.19 (MD 0.19, 95% CI 0.11 to 0.26, *p* < 0.00001, I^2^ = 81%), as shown in Figure 5A. The intergroup difference in ABI was 0.19, which exceeded the minimal clinically important difference (MCID) threshold of 0.085 (Xie et al., 2019), indicating that the difference in ABI has clinical significance. Furthermore, the sensitivity analysis revealed that the heterogeneity in ABI was primarily attributed to the study by Zhou et al., which may be due to their participants having a relatively short disease duration of only 4.7 years. After excluding the study by Zhou et al., the statistical significance of ABI remained (MD 0.22, 95% CI 0.17 to 0.28, *p* < 0.00001, I^2^ = 49%), suggesting that the results of the meta-analysis on ABI are robust.

##### 3.4.2.2 Common peroneal nerve MNCV

The meta-analysis of common peroneal nerve MNCV included four studies involving 265 participants. The results indicated that, compared to warm water foot baths, Chinese herbal foot baths significantly increased common peroneal nerve MNCV by 4.09 m/s (MD 4.09, 95% CI 2.41 to 5.77, *p* < 0.00001, I^2^ = 55%), as shown in Figure 5B. The intergroup difference in MNCV was 4.09 m/s, which exceeded the MCID threshold of 1.15 m/s (Huang et al., 2024), indicating that the difference in MNCV has clinical significance. Furthermore, the sensitivity analysis revealed that the heterogeneity in common peroneal nerve MNCV was primarily attributed to the study by Chen et al., which may be due to their participants having a relatively long disease duration of 9.2 years. After excluding the study by Chen et al., the statistical significance of common peroneal nerve MNCV remained (MD 4.81, 95% CI 3.46 to 6.16, *p* < 0.00001, I^2^ = 0%), suggesting that the results of the meta-analysis on common peroneal nerve MNCV are robust.

##### 3.4.2.3 Common peroneal nerve SNCV

The meta-analysis of common peroneal nerve SNCV included two studies involving 154 participants. The results indicated that, compared to warm water foot baths, Chinese herbal foot baths significantly increased common peroneal nerve SNCV by 3.83 m/s (MD 3.83, 95% CI 2.48 to 5.17, *p* < 0.00001, I^2^ = 0%), as shown in Figure 5C. The intergroup difference in SNCV was 3.83 m/s, which exceeded the MCID threshold of 0.74 m/s (Huang et al., 2024), indicating that the difference in SNCV has clinical significance. Furthermore, the sensitivity analysis confirmed the robust findings regarding common peroneal nerve SNCV.

#### 3.4.3 Blood glucose levels

##### 3.4.3.1 HbA1c

**FIGURE 6:**
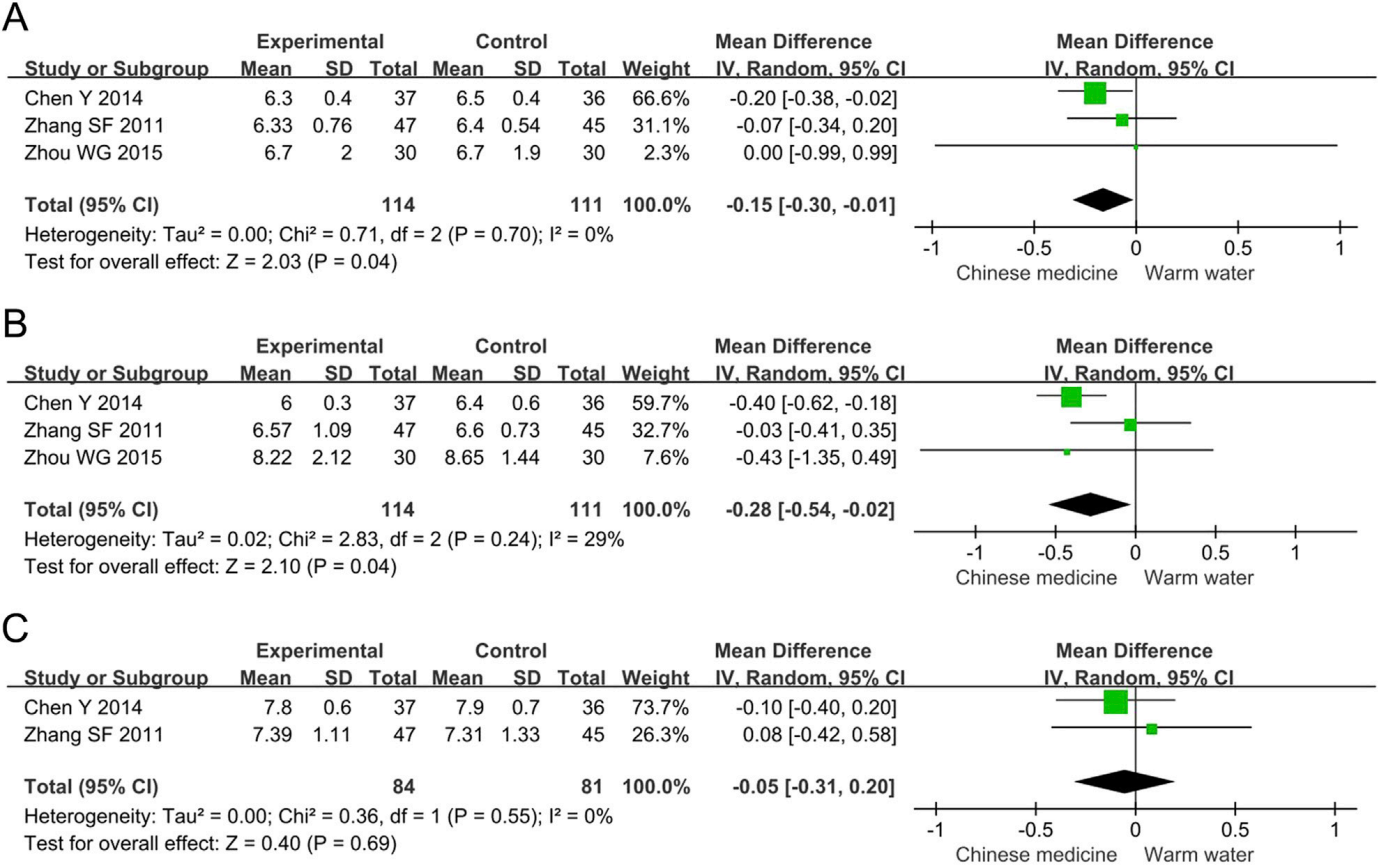
Forest plots of meta-analysis on the blood glucose levels: **(A)** HbA1c; **(B)** FBG; **(C)** 2h PBG. HbA1c, hemoglobin A1c; FBG, fasting blood glucose; PBG, postprandial blood glucose.

The meta-analysis of HbA1c included three studies involving 225 participants. The results indicated that, compared to warm water foot baths, Chinese herbal foot baths significantly reduced HbA1c levels by 0.15% (MD -0.15, 95% CI -0.30 to −0.01, *p* = 0.04, I^2^ = 0%), as shown in Figure 6A. However, the intergroup difference in HbA1c was 0.15%, which did not reach the MCID threshold of 0.5% (Goldenberg et al., 2021), suggesting that the difference in HbA1c lacks clinical significance. Furthermore, the sensitivity analysis revealed that after excluding the study by Chen et al., the statistical significance of HbA1c was lost (MD -0.07, 95% CI -0.32 to 0.19, *p* = 0.62, I^2^ = 0%), indicating that the results of the meta-analysis on HbA1c are not robust.

##### 3.4.3.2 FBG

The meta-analysis of FBG included three studies involving 225 participants. The results indicated that, compared to warm water foot baths, Chinese herbal foot baths significantly reduced FBG levels by 0.28 mmol/L (MD -0.28, 95% CI -0.54 to −0.02, *p* = 0.001, I^2^ = 29%), as shown in Figure 6B. However, the intergroup difference in FBG was 0.28 mmol/L, which did not reach the MCID threshold of 1.6 mmol/L (Goldenberg et al., 2021), suggesting that the difference in FBG lacks clinical significance. Furthermore, the sensitivity analysis revealed that the heterogeneity in FBG was primarily attributed to the study by Chen et al., which may be due to their participants having a relatively long disease duration of 9.2 years. After excluding the study by Chen et al., FBG lost statistical significance (MD -0.09, 95% CI -0.44 to 0.26, *p* = 0.62, I^2^ = 0%), indicating that the meta-analysis results of FBG are not robust.

##### 3.4.3.3 2h PBG

The meta-analysis of 2h PBG included two studies involving 165 participants. The results showed no statistically significant difference in the 2h PBG levels between the two groups (MD -0.05, 95% CI -0.31 to 0.20, *p* = 0.69, I^2^ = 0%), as shown in Figure 6C. The sensitivity analysis confirmed the robust findings regarding 2h PBG.

#### 3.4.4 Blood lipid levels

##### 3.4.4.1 TC

**FIGURE 7:**
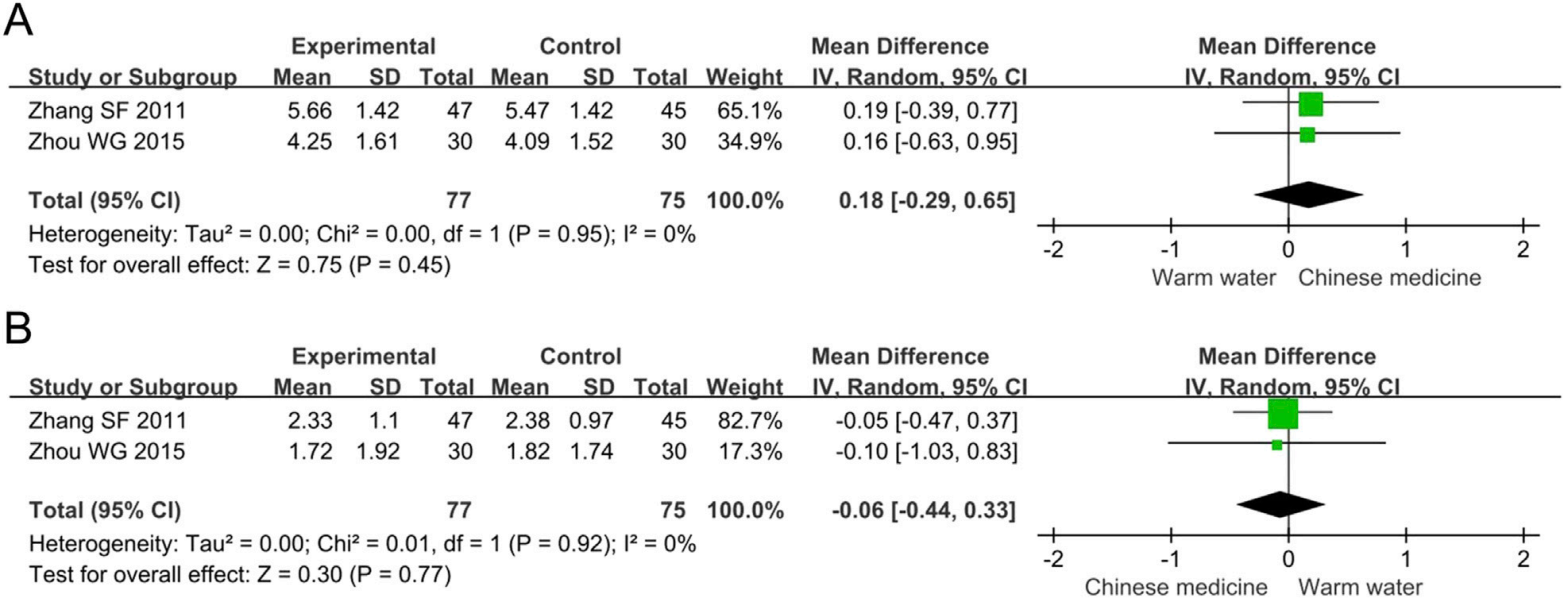
Forest plots of meta-analysis on the blood lipid levels: **(A)** TC; **(B)** TG. TC, total cholesterol; TG, triglycerides.

The meta-analysis of TC included two studies involving 152 participants. The results showed no statistically significant difference in the TC levels between the two groups (MD 0.18, 95% CI -0.29 to 0.65, *p* = 0.45, I^2^ = 0%), as shown in Figure 7A. The sensitivity analysis confirmed the robust findings regarding TC.

##### 3.4.4.2 TG

The meta-analysis of TG included two studies involving 152 participants. The results showed no statistically significant difference in the TG levels between the two groups (MD -0.06, 95% CI -0.44 to 0.33, *p* = 0.77, I^2^ = 0%), as shown in Figure 7B. The sensitivity analysis confirmed the robust findings regarding TG.

#### 3.4.5 Safety outcomes

The meta-analysis of adverse events incorporated three studies with a total of 239 participants. The incidence of adverse events was 0 in both the experimental group (n = 129) and the control group (n = 110). However, due to the complete absence of adverse events in both the experimental and control groups, the Peto-OR could not be computed. This is because the calculation formula for the Peto-OR involves elements that become undefined when the event counts are zero, which is a limitation of this statistical method in such scenarios. Therefore, while the current data suggest that the Chinese herbal foot bath did not result in any additional adverse events, further research with larger sample sizes is needed to support more robust statistical analyses.

### 3.5 Meta-regression analysis

The I^2^ statistics indicated a high level of heterogeneity for the results of ABI and MNCV, while moderate heterogeneity was observed for HbA1c. Consequently, a meta-regression analysis was employed to investigate the sources and impacts of heterogeneity for ABI, MNCV, and HbA1c. However, due to the inclusion of fewer than ten studies for these outcomes, the meta-regression analysis was limited and could not be conducted. Additionally, considering that sensitivity analyses had already identified potential sources of heterogeneity for ABI, MNCV, and HbA1c, a meta-regression was not performed in this study.

### 3.6 Subgroup analysis

Subgroup analysis, using the clinical effective rate as the outcome measure, investigated the impact of factors such as Wagner grade, treatment frequency, treatment duration, and the composition and dosage of Chinese herbal medicine on the efficacy of Chinese herbal foot baths in the treatment of diabetic foot, as shown in Table 2.

**TABLE 2:** Subgroup analyses of Chinese herbal foot-baths for diabetic foot with Wagner grade of 0 or 1.

Subject	Subgroup	Experimental group	Control group	RR (95%CI)	*p* value
Wagner	Grade 0	233/252	162/252	1.44 (1.30, 1.58)	<0.00001
	Grade 1	56/62	29/44	1.37 (1.09, 1.72)	0.007
	Grade 0-1	102/115	66/115	1.54 (1.30, 1.82)	<0.0001
Frequency	30 min bid	47/55	29/56	1.64 (1.25, 2.15)	0.0004
	30 min qd	186/200	128/201	1.46 (1.31, 1.63)	<0.00001
	20 min bid	26/28	17/28	1.53 (1.12, 2.10)	0.008
	20 min qd	91/97	56/79	1.33 (1.15, 1.55)	0.0002
	20 min q2d	39/47	23/45	1.62 (1.19, 2.22)	0.002
Course of treatment	≤ 30 days	186/198	132/199	1.41 (1.27, 1.57)	<0.00001
	> 30 days	203/229	121/210	1.53 (1.35, 1.73)	<0.00001

For the Wagner grade, the statistical threshold corrected by Bonferroni method is *p* < 0.0167. The reusults showed that Chinese herbal foot baths significantly improved the clinical effective rates of diabetic foot patients with Grade 0 (RR 1.39, 95% CI 1.26 to 1.53, *p* < 0.00001), Grade 1 (RR 1.37, 95% CI 1.09 to 1.72, *p* = 0.007), and Grades 0–1 (RR 1.47, 95% CI 1.26 to 1.72, *p* < 0.0001).

Regarding treatment frequency, the statistical threshold corrected by Bonferroni method is *p* < 0.01. The following results were observed: 30 min bid (RR 1.61, 95% CI 1.20 to 2.15, *p* = 0.001), 30 min qd (RR 1.41, 95% CI 1.26 to 1.58, *p* < 0.00001), 20 min bid (RR 1.53, 95% CI 1.12 to 2.10, *p* = 0.008), 20 min qd (RR 1.32, 95% CI 1.14 to 1.53, *p* = 0.0001), and 20 min q2d (RR 1.62, 95% CI 1.19 to 2.22, *p* = 0.002) of the Chinese herbal foot bath all significantly improved the clinical effective rate.

In terms of treatment duration, the statistical threshold corrected by Bonferroni method is *p* < 0.025. The results suggested that both ≤30 days (RR 1.39, 95% CI 1.25 to 1.54, *p* < 0.00001) and >30 days (RR 1.48, 95% CI 1.29 to 1.70, *p* < 0.00001) of the Chinese herbal foot bath significantly improved the clinical effective rate.

However, due to the variability in the composition and dosage of the Chinese herbal formulas used for the foot bath, it was not possible to conduct a subgroup analysis based on the herbal composition or formula.

### 3.7 Trial sequential analysis

**FIGURE 8:**
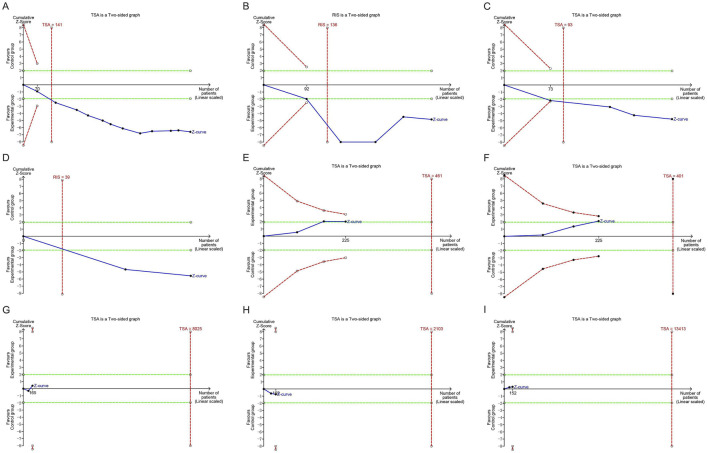
Trial sequential analyses of efficacy outcomes: **(A)** Clinical effective rate; **(B)** ABI; **(C)** Common peroneal nerve MNCV; **(D)** Common peroneal nerve SNCV; **(E)** HbA1c; **(F)** FBG; **(G)** 2h PBG; **(H)** TC; **(I)** TG. ABI, ankle-brachial index; MNCV, motor nerve conduction velocity; SNCV, sensory nerve conduction velocity; HbA1c, hemoglobin A1c; FBG, fasting blood glucose; PBG, postprandial blood glucose; TC, total cholesterol; TG, triglycerides.

The TSA demonstrated that the cumulative Z-curve for the clinical effective rate, ABI, common peroneal nerve MNCV, and common peroneal nerve SNCV crossed the monitoring boundaries, indicating that the meta-analysis results for these outcomes have reached the required sample size and are conclusive. In contrast, the cumulative Z-curve for HbA1c, FBG, 2h PBG, TC, and TG did not cross the monitoring boundaries, suggesting that more studies are needed to further evaluate and validate these findings, as shown in Figure 8.

### 3.8 Publication bias

**FIGURE 9:**
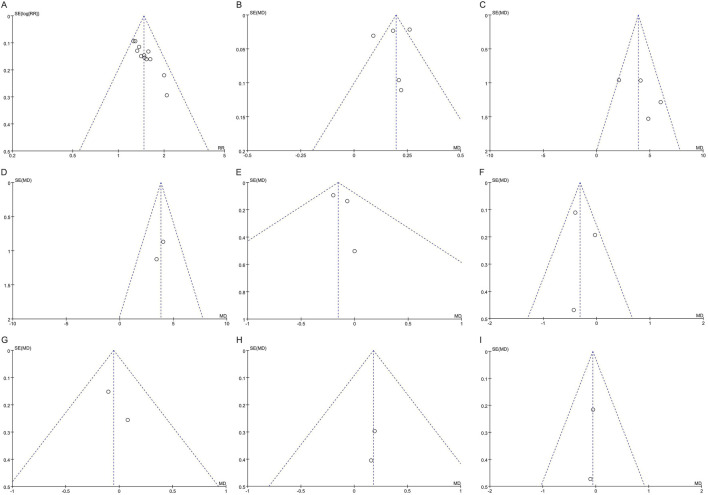
Funnel plots of publication bias: **(A)** Clinical effective rate; **(B)** ABI; **(C)** Common peroneal nerve MNCV; **(D)** Common peroneal nerve SNCV; **(E)** HbA1c; **(F)** FBG; **(G)** 2h PBG; **(H)** TC; **(I)** TG. ABI, ankle-brachial index; MNCV, motor nerve conduction velocity; SNCV, sensory nerve conduction velocity; HbA1c, hemoglobin A1c; FBG, fasting blood glucose; PBG, postprandial blood glucose; TC, total cholesterol; TG, triglycerides.

Funnel plots for ABI, common peroneal nerve SNCV, 2h PBG, TC, and TG exhibited symmetric scatter distributions on both sides, indicating no evidence of publication bias. In contrast, the funnel plots for the clinical effective rate, common peroneal nerve MNCV, HbA1c, and FBG displayed asymmetric scatter distributions, suggesting potential publication bias, as shown in Figure 9.

Furthermore, Egger’s tests indicated no significant potential publication bias for ABI (*p* = 0.806), common peroneal nerve MNCV (*p* = 0.328), HbA1c (*p* = 0.553), and FBG (*p* = 0.793), suggesting that the publication bias observed in the funnel plots is not severe. However, the Egger’s test for the clinical effective rate showed evidence of potential publication bias (*p* < 0.001). It is important to note that Egger’s tests could not be performed for common peroneal nerve SNCV, 2-h PBG, TC, and TG, as these analyses included only two studies.

### 3.9 Certainty of evidence

The GRADE approach indicated that the certainty of evidence for the clinical effective rate, common peroneal nerve SNCV, HbA1c, FBG, 2h PBG, TC, TG, and adverse events was low. In contrast, the certainty of evidence for ABI and common peroneal nerve MNCV was very low, as shown in Table 3.

**TABLE 3:** Certainty of evidence.

Outcome	Risk of bias	Inconsistency	Indirectness	Imprecision	Others	RR/MD (95% CI)	Certainty of evidence
Clinical effective rate	Serious	None	None	None	Publication bias	−9.41 (−18.78, −0.05)	Low
ABI	Serious	Serious	None	Serious	None	−0.93 (−5.70, 3.84)	Very Low
Common peroneal nerve MNCV	Serious	Serious	None	Serious	None	3.42 (1.53, 5.32)	Very Low
Common peroneal nerve SNCV	Serious	None	None	Serious	None	−3.21 (−13.49, 7.06)	Low
HbA1c	Serious	None	None	Serious	None	−12.28 (−31.85, 7.29)	Low
FBG	Serious	None	None	Serious	None	−20.39 (−32.00, −8.79)	Low
2h PBG	Serious	None	None	Serious	None	−2.60 (−5.43, 0.22)	Low
TC	Serious	None	None	Serious	None	−0.31 (−1.46, 0.84)	Low
TG	Serious	None	None	Serious	None	0.00 (−0.01, 0.01)	Low
Adverse events	Serious	None	None	Serious	None	2.00 (0.20, 20.49)	Low

RR, risk ratio; MD, mean difference; CI, confidence interval; ABI, ankle-brachial index; MNCV, motor nerve conduction velocity; SNCV, sensory nerve conduction velocity; HbA1c, hemoglobin A1c; FBG, fasting blood glucose; PBG, postprandial blood glucose; TC, total cholesterol; TG, triglycerides.

### 3.10 Data mining

The frequency analysis included 13 regimens of Chinese herbal foot baths and 61 Chinese herbal medicines. Among these, the frequencies of [*Cinnamomum cassia* Presl] (69.23%), [*Conioselinum anthriscoides* ‘Chuanxiong’] (46.15%) [*Carthamus tinctorius* L.], (46.15%) [*Prunus persica* (L.), Batsch] (46.15%), [*Angelica sinensis* (Oliv.) Diels] (38.46%), [*Asarum heterotropoides* F. Schmidt] (38.46%), [*Salvia miltiorrhiza* Bunge] (30.77%), and [*Paeonia lactiflora* Pall.] (30.77%) all exceeded 30%, which were considered as high frequency herbs for diabetic foot with Wagner grade of 0 or 1, as shown in Table 4.

**TABLE 4:** Frequency analyses of Chinese herbal foot-baths for diabetic foot with Wagner grade of 0 or 1.

Rank	Chinese herbal	Frequency (n/%)
1	[*Cinnamomum cassia* Presl]	9 (69.23)
2	[*Conioselinum anthriscoides* ‘Chuanxiong’]	6 (46.15)
3	[*Carthamus tinctorius* L.]	6 (46.15)
4	[*Prunus persica* (L.) Batsch]	6 (46.15)
5	[*Angelica sinensis* (Oliv.) Diels]	5 (38.46)
6	[*Asarum heterotropoides* F.Schmidt]	5 (38.46)
7	[*Salvia miltiorrhiza* Bunge]	4 (30.77)
8	[*Paeonia lactiflora* Pall.]	4 (30.77)

**FIGURE 10:**
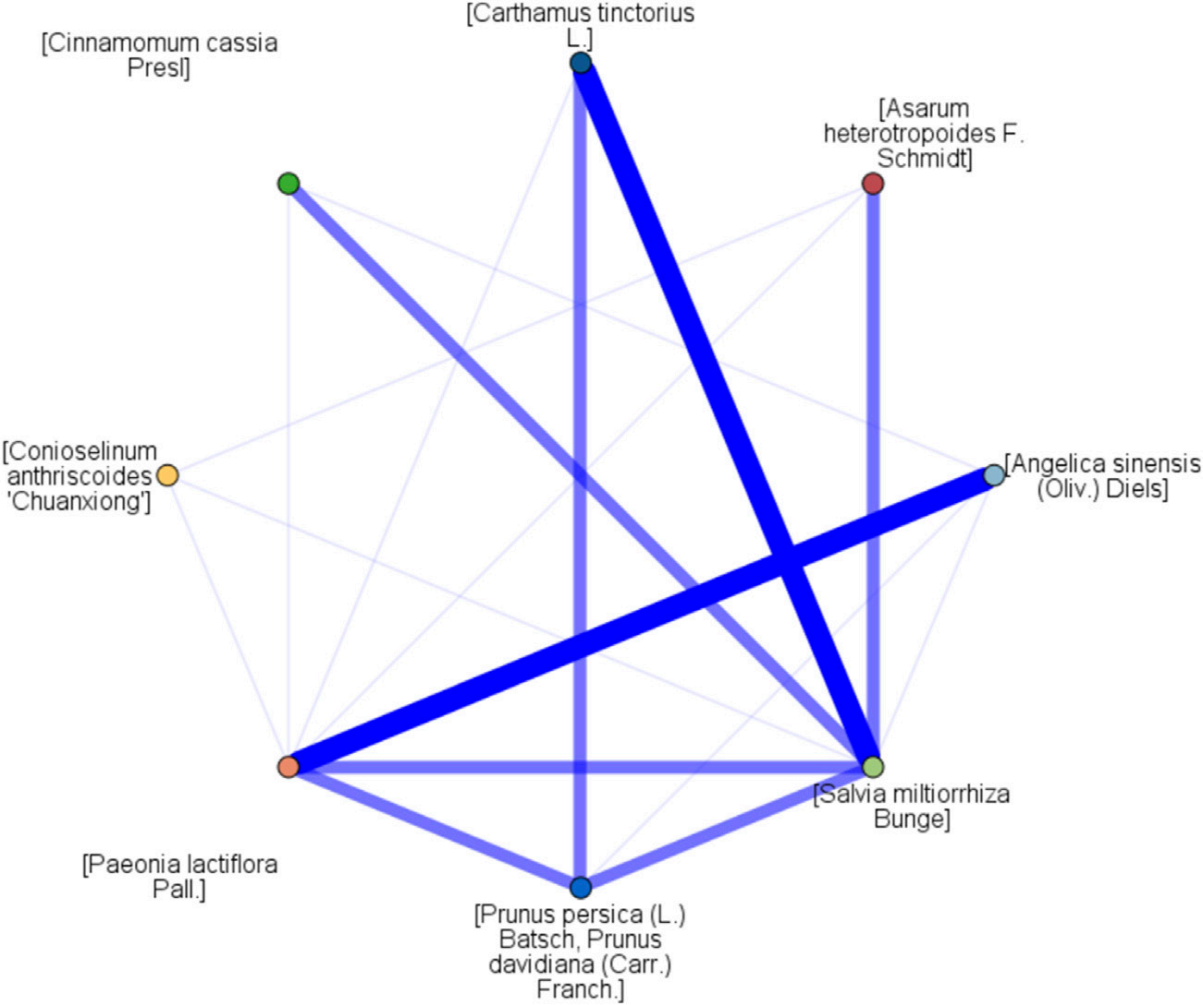
Association network of high-frequency herbs.

The network diagram of association rule analysis is shown in Figure 10. The association rule analysis reported 11 results and seven combinations of Chinese herbal medicine, as recorded in Table 5. The combinations of “[*Cinnamomum cassia* Presl] - [*Conioselinum anthriscoides* ‘Chuanxiong’]”, “[*Paeonia lactiflora* Pall.] - [*Angelica sinensis* (Oliv.) Diels]”, “[*Prunus persica* (L.) Batsch] - [*Carthamus tinctorius* L.]”, “[*Cinnamomum cassia* Presl] - [*Carthamus tinctorius* L.]”, “[*Cinnamomum cassia* Presl] - [*Prunus persica* (L.) Batsch]”, “[*Cinnamomum cassia* Presl] - [*Asarum heterotropoides* F. Schmidt]”, and “[*Cinnamomum cassia* Presl] - [*Carthamus tinctorius* L.] - [*Prunus persica* (L.) Batsch]” emerged as key candidate herbs combinations for foot baths. These combinations encompassed the key candidate herbs such as [*Cinnamomum cassia* Presl], [*Conioselinum anthriscoides* ‘Chuanxiong’], [*Paeonia lactiflora* Pall.], [*Angelica sinensis* (Oliv.) Diels] [*Prunus persica* (L.), Batsch], [*Carthamus tinctorius* L.], and [*Asarum heterotropoides* F. Schmidt].

**TABLE 5:** Association rule analyses of Chinese herbal foot-baths for diabetic foot with Wagner grade of 0 or 1.

Preceding paragraph	Behind paragraph	Support/%	Confidence/%	Lift
[*Cinnamomum cassia* Presl]	[*Conioselinum anthriscoides* ‘Chuanxiong’]	46.15	83.33	1.20
[*Paeonia lactiflora* Pall.]	[*Angelica sinensis* (Oliv.) Diels]	38.46	80.00	2.60
[*Prunus persica* (L.) Batsch]	[*Carthamus tinctorius* L.]	38.46	80.00	2.08
[*Carthamus tinctorius* L.]	[*Prunus persica* (L.) Batsch]	38.46	80.00	2.08
[*Cinnamomum cassia* Presl]	[*Carthamus tinctorius* L.]	38.46	80.00	1.16
[*Cinnamomum cassia* Presl]	[*Prunus persica* (L.) Batsch]	38.46	80.00	1.16
[*Angelica sinensis* (Oliv.) Diels]	[*Paeonia lactiflora* Pall.]	30.77	100.00	2.60
[*Cinnamomum cassia* Presl]	[*Asarum heterotropoides* F.Schmidt]	30.77	100.00	1.44
[*Cinnamomum cassia* Presl]	[*Carthamus tinctorius* L.] and [*Prunus persica* (L.) Batsch]	30.77	100.00	1.44
[*Prunus persica* (L.) Batsch]	[*Carthamus tinctorius* L.] and [*Cinnamomum cassia* Presl]	30.77	100.00	2.60
[*Carthamus tinctorius* L.]	[*Prunus persica* (L.) Batsch] and [*Cinnamomum cassia* Presl]	30.77	100.00	2.60

## 4 Discussion

### 4.1 Research significance and main findings

The potential of Chinese herbal foot baths in the treatment of diabetic foot has received more and more attention. To the best of our knowledge, this study is the first meta-analysis and data mining of Chinese herbal foot baths in the treatment of diabetic foot with Wagner grade of 0 or 1. This meta-analysis indicates that, compared to warm water foot baths, Chinese herbal foot baths significantly improve the clinical effective rate, ABI, and MNCV and SNCV of the common peroneal nerve, with these results being robust. Although the meta-analysis showed a slight reduction in FBG and HbA1c levels with Chinese herbal foot baths, these outcomes were not robust. Additionally, the analysis supports that Chinese herbal foot baths have no significant impact on 2h PBG, TC, or TG levels, and do not increase the incidence of adverse events.

### 4.2 Evaluation of efficacy

The meta-analysis revealed that Chinese herbal foot baths, compared to the warm water foot baths, improved the clinical effective rate by 42%, the ABI by 0.19, the common peroneal nerve MNCV by 4.09 m/s, and the common peroneal nerve SNCV by 3.83 m/s. These findings were confirmed to be robust and conclusive through sensitivity analysis and TSA. The clinical effective rate refers to the proportion of participants rated as effective relative to the total number of participants, used to evaluate the improvement of diabetic foot-related symptoms. These symptoms included numbness, tingling, cold skin, dullness or loss of sensation, and superficial ulcers on the foot, which are the result of a combination of peripheral vascular and neuropathy in the diabetic foot. The significant improvement in clinical effective rate suggests that Chinese medicine foot baths can effectively relieve clinical symptoms in patients with diabetic foot, thereby reducing their pain and overall disease burden. ABI serves as an assessment tool for evaluating the severity of lower limb arterial stenosis, with values below 0.9 indicating possible stenosis or obstruction of lower limb arteries. Given that diabetic patients have a higher risk of lower extremity arterial stenosis or obstruction, ABI represents an important indicator to evaluate the blood flow and vascular function of their feet (Brito-Zurita et al., 2013). The common peroneal nerve MNCV and SNCV are indicators for evaluating the nerve function of the lower limbs and are of great significance in evaluating the degree of neuropathy in diabetic foot (Lawrence and Locke, 1961). The benefits observed in ABI, MNCV, and SNCV indicate that Chinese herbal foot baths have the effect of improving peripheral vascular and neuropathy, and it may be the direct mechanism by which it alleviates symptoms and signs related to diabetic foot.

The results of meta-analysis also indicated that Chinese herbal foot baths reduced HbA1c and FBG compared with warm water foot baths, while 2h PBG, TC and TG were all equivalent. However, the Chinese herbal foot bath only reduced HbA1c by 0.15% and FBG by 0.28 mmol/L, which did not meet the MCID, indicating that these differences are not clinically significant. Additionally, sensitivity analysis revealed that the benefit of Chinese herbal foot baths in blood glucose levels was driven by the study of Chen and Zhao (2014). After removing this study, the pooled results showed that there was no significant difference in HbA1c (MD -0.07, 95% CI -0.32 to 0.19, *p* = 0.62) and FBG (MD -0.09, 95% CI -0.44 to 0.26, *p* = 0.62) between two groups. This finding underscores the fragility of the observed glycemic benefit. Moreover, TSA indicated that the cumulative sample sizes for HbA1c, FBG, 2h PBG, TC, and TG did not reach the required thresholds, highlighting the need for further studies to investigate the effects of Chinese herbal foot baths on blood glucose and lipid levels.

The subgroup analysis revealed that the clinical effective rate of Chinese herbal foot baths for treating diabetic foot with grade 0, grade 1, and grade 0–1 was significantly higher than that of warm water foot baths. This indicates that Chinese herbal foot baths offer added benefits for diabetic foot patients with Wagner grade of 0 or 1. From the perspective of treatment frequency, regimens such as 30 min bid, 30 min qd, 20 min bid, 20 min qd, and 20 min q2d all showed significantly higher clinical effective rates than warm water foot baths. This suggests that Chinese herbal foot baths administered within this range—from 20 min q2d to 30 min bid—consistently demonstrate superior efficacy. In terms of treatment duration, both short-term (≤30 days) and long-term (>30 days) use of Chinese herbal foot baths were associated with better clinical outcomes compared to warm water foot baths. These findings suggest that the therapeutic benefits of Chinese herbal foot baths are not limited by the severity of the condition, treatment frequency, or duration, allowing clinicians to tailor regimens based on patient tolerance and clinical need.

### 4.3 Evaluation of safety

Our meta-analysis found that among the limited number of studies reporting safety outcomes, 129 patients with diabetic foot who received Chinese herbal foot baths and 110 who received warm water foot baths experienced no treatment-related adverse events. This suggests that Chinese herbal foot baths may not increase the risk of additional adverse events. As a form of topical therapy, Chinese herbal foot baths theoretically reduce risks associated with oral absorption and hepatic or renal metabolism. Prior systematic reviews in other conditions, such as dysmenorrhea and hypertension, have also reported no additional adverse events, indicating a potentially favorable safety profile (Tian et al., 2024; Wu T. et al., 2024). However, only a small number of included studies reported on adverse events, and most lacked detailed safety data. As such, current evidence remains insufficient to draw firm conclusions regarding the safety of Chinese herbal foot baths for diabetic foot. Further high-quality studies with rigorous safety monitoring and standardized adverse event reporting are needed to better evaluate their safety profile.

### 4.4 Mechanism of key candidate herbs

The data mining revealed that [*Cinnamomum cassia* Presl], [*Conioselinum anthriscoides* ‘Chuanxiong'], [*Paeonia lactiflora* Pall.], [*Angelica sinensis* (Oliv.) Diels] [*Prunus persica* (L.), Batsch], [*Carthamus tinctorius* L.], and [*Asarum heterotropoides* F. Schmidt] were the key candidate herbs for foot baths in treating diabetic foot with Wagner grades of 0 or 1.

Cinnamaldehyde, the main active compound of [*Cinnamomum cassia* Presl], exerts neuroprotective effects against diabetes-related neuronal damage by inhibiting advanced glycation end-products (AGE)-induced abnormal cell cycle re-entry and apoptosis, thereby preserving neuronal cells and maintaining their terminally differentiated state (Wu Y. et al., 2024). Another significant compound from [*Cinnamomum cassia* Presl], eugenol, promotes angiogenesis in diabetic model mice and accelerates wound healing in diabetic wounds (Han et al., 2024). Additionally, Z-ligustilide extracted from [*Conioselinum anthriscoides* ‘Chuanxiong'] can reduce inflammation and oxidative stress by activating Nuclear factor erythroid 2-related factor 2 (NRF2), indicating its potential for treating diabetic foot (Yang et al., 2018; Qi et al., 2024). The role of [*Paeonia lactiflora* Pall.] in diabetic neuropathy has garnered increasing attention due to their antinociceptive, anti-inflammatory, antioxidant, and anti-apoptotic activities (Wiegand et al., 2024). Its major compound, paeoniflorin, promotes the transition of macrophages from the pro-inflammatory M1 phenotype to the anti-inflammatory/pro-healing M2 phenotype, thereby accelerating wound healing (Yang et al., 2021). Angelica sinensis polysaccharide, a key component of [*Angelica sinensis* (Oliv.) Diels], exhibits hypoglycemic, hypolipidemic, anti-inflammatory, and hepatoprotective effects by improving insulin sensitivity, enhancing glycogen synthesis, modulating adipokine secretion, and inhibiting apoptosis in diabetic models (Wang et al., 2015; 2016). Amygdalin, the main component of [*Prunus persica* (L.) Batsch], protects against high-glucose-induced ferroptosis and oxidative stress in retinal endothelial cells by activating the NRF2/antioxidant response element (ARE) signaling pathway, highlighting its therapeutic potential in alleviating diabetes-related microvascular damage (Li S. et al., 2023). Hydroxysafflor yellow A, an active component of [*Carthamus tinctorius* L.], protects pancreatic β-cells by alleviating oxidative stress-induced damage in T2DM rat models (Xue et al., 2021; Alshareef et al., 2024). Asarone, an extract from [*Asarum heterotropoides* F. Schmidt], improves insulin levels and mitigates inflammation, fibrosis, and carcinogenesis, thereby reducing the risk of diabetic complications (Das et al., 2019). In summary, these herbs and their active compounds demonstrate potential in managing diabetic foot and related complications through various mechanisms, including anti-inflammatory, antioxidant, and neuroprotective effects.

### 4.5 Scheme analysis of foot bath

Apart from the composition of Chinese herbal medicine, the regimen for Chinese herbal foot baths involves factors such as the temperature and depth of the medicinal solution, the soaking duration, the commencement time, and the treatment duration. (i) The temperature of the medicinal solution. The 2011 International Diabetic Foot Working Group Guidelines for the Disposition and Prevention of the Diabetic Foot suggest that the water temperature of foot baths should be lower than 40°C, with the recommendation to test the water temperature by hand and adjust accordingly until it feels suitable (Xu and Li, 2013). The clinical study by Hu Y and colleagues (Xu and Li, 2013) demonstrated that the effects of Chinese herbal foot baths at 36°C, 38°C, and 40°C were comparable, indicating that the range of 36°C–40°C is suitable for treating diabetic foot. In practice, the temperature of the medicinal solution should be flexibly adjusted within the range of 36°C–40°C according to the specific conditions of patients. (ii) The depth of the medicinal solution. A related study showed that the effect of Chinese herbal foot baths when the depth of the medicinal solution reached 20 cm above the ankle was significantly better than that of 10 cm above the ankle and just submerging the ankle (Hu et al., 2013), suggesting that 20 cm above the ankle may be the optimal depth of the medicinal solution. (iii) The soaking duration. A literature survey on the duration of foot baths showed that the optimal soaking time for foot baths was 20–30 min (Zheng, 2012). A subsequent clinical trial found that the group immersed for 30 min in Chinese herbal foot baths significantly improved the ABI and toe-brachial index better than the 25-min and 20-min groups (Hu et al., 2013), suggesting that 30 min may be the optimal soaking time. (iv) The commencement time. Hu et al. recommended arranging Chinese herbal foot baths between 19:00 and 21:00 because foot baths at this time of day help patients relax and improve sleep quality (Hu et al., 2013). (v) The treatment duration. The included studies showed that Chinese herbal foot baths from 14 days to 90 days were significantly more effective than warm water foot baths, so the duration of Chinese herbal foot baths is recommended to be set at 14 days and above.

In conclusion, we recommend the following regimen for treating diabetic foot with Wagner grade of 0 or 1 using Chinese herbal foot-baths: key medicinal herbs should be added to 4,000 mL of water and boiled for 30 min to prepare the therapeutic solution. The solution should be maintained at a temperature of 36°C–40°C and should reach a depth of approximately 20 cm above the ankle. Each soaking session should last for 30 min, preferably between 19:00 and 21:00. The treatment should be continued for at least 14 days to ensure optimal therapeutic outcomes. It is important to emphasize that the recommended herbal formula is exploratory in nature, derived from preliminary data mining and literature review. While the findings of the current study are encouraging, the pharmacological effects of the formulation have not been rigorously validated. Moreover, the considerable variability in herbal formulations across clinical settings may influence their therapeutic outcomes. As such, these recommendations should not be interpreted as definitive clinical guidance but rather as hypothesis-generating insights. Rigorous validation through high-quality RCTs is necessary to establish both the efficacy and safety of the proposed regimen. Future research should also aim to standardize herbal compositions to improve the reproducibility and reliability of clinical outcomes.

### 4.6 Limitations and prospects

This study has several notable limitations. First, all included studies did not report details of allocation concealment, which increases the risk of selective bias. More seriously, the lack of blinding in the majority of the included studies (10 out of 13) raises significant concerns regarding performance bias, which may undermine the reliability of the meta-analytic outcomes. These factors contribute to a high risk of underreporting and undermine confidence in the reported results, particularly by increasing the risk of false positives in subjective outcomes such as the clinical effective rate. Second, while the focus on patients with Wagner Grade 0–1 diabetic foot enhances internal validity, it restricts the external generalizability of the findings. Third, all included studies were conducted in China with exclusively Chinese participants, which raises concerns about ethnogeographic bias and the applicability of the results to non-Asian populations. Fourth, the adverse events were infrequently reported, with only three studies documenting zero events. This limitation restricts our ability to draw robust conclusions about the safety of the intervention. Fifth, due to substantial variations in herbal compositions across the included studies, we were unable to conduct subgroup analyses or meta-regression to further evaluate the associations between specific herbs and clinical outcomes, as well as herb-drug interactions, standardization, and pharmacokinetic properties. Sixth, our recommended herbal formula was derived from data mining and literature review, and while its pharmacological mechanisms and optimal dosage have not been rigorously validated. Furthermore, the cultural acceptance and regulatory barriers associated with implementing herbal foot baths therapy globally, along with the heterogeneity in herbal combinations even within the included studies, complicate the interpretation of the findings.

Future research should aim to address these limitations comprehensively. To improve methodological quality, future studies should incorporate rigorous designs with clear randomization, allocation concealment, and blinding procedures to reduce the risks of bias. Establishing research centers across different countries and diverse populations will help evaluate the efficacy and safety of Chinese herbal foot baths therapy beyond the current ethnogeographic context, enhancing external validity. Multi-center clinical trials using standardized herbal formulations are essential to confirm the therapeutic benefits and to facilitate reproducibility. Moreover, improved reporting of adverse events and safety data is crucial for a comprehensive safety profile. Standardization of herbal components and dosing regimens should be prioritized to minimize heterogeneity and ensure consistent results. Additionally, mechanistic studies are needed to elucidate the pharmacological actions underlying observed clinical effects, which could improve acceptance and integration into broader healthcare systems. Addressing cultural acceptance and navigating regulatory barriers at an international level will be vital for the global implementation of herbal foot baths therapy. Collectively, these efforts will help overcome current methodological, safety, and applicability limitations and advance the evidence base for this traditional intervention.

## 5 Conclusion

Chinese herbal foot baths appear to improve clinical symptoms as well as vascular and nerve functions in diabetic foot patients with Wagner grades 0 or 1, and they may not increase the incidence of adverse events. The foot baths regimen comprising [*Cinnamomum cassia* Presl], [*Conioselinum anthriscoides* ‘Chuanxiong'], [*Paeonia lactiflora* Pall.], [*Angelica sinensis* (Oliv.) Diels] [*Prunus persica* (L.), Batsch], [*Carthamus tinctorius* L.], and [*Asarum heterotropoides* F. Schmidt] is expected to be a complementary treatment for diabetic foot. However, the above herbal formula is exploratory in nature and requires further validation through clinical trials and pharmacological studies. Additionally, due to the limited evidence available, future research should aim to clarify the effects of Chinese herbal foot baths on blood glucose levels, blood lipids, and safety outcomes.

## Data Availability

The original contributions presented in the study are included in the article/Supplementary Material, further inquiries can be directed to the corresponding authors.

## Abbreviations

ABI, Ankle-brachial index; CI, Confidence interval; FBG, Fasting blood glucose; HbA1c, Hemoglobin A1c; MD, Mean difference; MNCV, Motor nerve conduction velocity; PBG, Postprandial blood glucose; PRISMA, Pref erred Reporting Items for Systematic reviews and Meta-Analyses; RR, Risk ratio; SNCV, Sensory nerve conduction velocity; TC, Total cholesterol; TG, Triglycerides.
